# Eye-Tracking Technology in Dentistry: A Review of Literature

**DOI:** 10.7759/cureus.55105

**Published:** 2024-02-27

**Authors:** Amani A Al Tuwirqi

**Affiliations:** 1 Pediatric Dentistry Department, Faculty of Dentistry, King Abdulaziz University, Jeddah, SAU

**Keywords:** gaze pattern, dental education, dentistry, eye tracking technology, eye tracking

## Abstract

This narrative review explores the integration of eye-tracking technology in dentistry, aiming to provide a comprehensive overview of its current applications and potential benefits. The review begins by elucidating the fundamental principles of eye tracking, encompassing the various eye-tracking methods and devices commonly used in dental research. It then delves into the role of eye tracking in dental education, where the technology offers a unique perspective on students' visual attention during training and skill acquisition. Moreover, the review examines how eye tracking can aid in assessing and improving dental practitioners' clinical performance, shedding light on areas of improvement and expertise. In patient care, the application of eye-tracking technology offers significant potential. By analyzing patients' gaze patterns and visual focus during dental procedures, clinicians can gain valuable insights into their experiences, identifying sources of anxiety and discomfort. This newfound understanding can pave the way for more patient-centric care and optimized treatment plans. The review also explores the application of eye-tracking technology in designing and evaluating dental interfaces and equipment. By assessing visual ergonomics and usability, researchers can develop user-friendly instruments that enhance dental professionals' workflow and efficiency. However, despite its promise, integrating eye tracking in dentistry is not without challenges. Technical limitations, data analysis complexities, and ethical considerations require careful attention to ensure this technology’s ethical and responsible use. In conclusion, this narrative review highlights the growing significance of eye-tracking technology in dentistry. Its applications span dental education, clinical practice, and patient care, holding immense potential to revolutionize how dental procedures are conducted, evaluated, and experienced. Nevertheless, further research and collaboration between dental professionals and eye-tracking experts are necessary to unlock the technology's benefits and ensure its seamless integration into dental practices.

## Introduction and background

Researchers have been using eye-tracking technology to understand how people perceive and interact with their environment for many years. Eye-tracking technology originated in the late 1800s and early 1900s when researchers started developing instruments to measure eye movements during reading and visual perception tasks. French ophthalmologist Louis Emile Javal invented the first eye tracker in 1879, which used a mirror to reflect an image of the eye onto a photographic plate [1].

In the following decades, eye tracking technology continued to evolve with the development of more sophisticated devices such as electro-oculography (EOG) and scleral search coil, enabling more accurate eye movement measurements. The advent of digital technology in the 1990s led to the development of video-based eye trackers that used infrared cameras to capture eye movements, which are still widely used today [2].

Eye tracking technology uses specialized hardware and software to monitor and analyze the movement and position of the eyes. There are different types of eye-tracking systems, including remote eye trackers and head-mounted eye trackers. The process involves calibration, where the user focuses on specific points to establish the relationship between eye movements and visual stimuli. The system detects and measures various types of eye movements, such as fixations, saccades, and smooth pursuits. The captured eye movement data is then analyzed to generate insights about visual attention, cognitive processes, and user behavior [3].

Eye tracking devices also provide gaze behavior analysis which examines the eye movement data to understand visual attention and cognitive processes. It analyzes fixations, saccades, and scanpaths to determine attention patterns. Also, areas of interest (AOIs) and heatmaps can be identified to visualize the important regions and attention distribution [3].

## Review

Eye tracking in dentistry

Eye-tracking technology has been extensively used in various fields, including psychology, marketing, and gaming. Recently, this technology has gained attention in the field of dentistry as it offers a noninvasive and accurate method of monitoring visual attention and eye movements during various tasks.

In dentistry, the use of eye-tracking technology has been explored in recent years to understand patient behavior, attention, and perception during clinical procedures. Eye-tracking technology in dentistry aims to improve the clinician's performance by providing real-time feedback on eye movements during dental procedures. This review explores the potential applications of eye-tracking technology in dentistry and its current status [4].

Several studies have been conducted to evaluate the application of eye tracking in dental treatment modalities, patients’ psychology and behavior, ergonomic evaluation of the dental operatory as well as dental education [5-8].

Dental Treatment Modalities

One of the applications of eye-tracking technology is to evaluate the treatment outcomes. Eye-tracking metrics can assess the effectiveness of different treatment modalities and techniques. For example, eye-tracking can be used to evaluate the effectiveness of local anesthesia applications during dental procedures. A study conducted by Kim et al. found that eye-tracking metrics were significantly correlated with the success rate of local anesthesia delivery [5]. Another study by Hofmaenner highlighted the potential of eye-tracking technology in improving patient safety and preventing adverse events during dental procedures [6].

Patients’ Psychology and Behavior

Several studies have used eye-tracking technology to investigate the behavior and perception of patients during dental procedures. They found that patients who perceived dental visual stimuli as threatening had considerably higher fixation duration times on the dental instruments than those who did not perceive the stimuli as threatening. Additionally, patients who reported higher levels of dental anxiety had longer fixation duration on the dental instruments than those with lower anxiety levels. This suggests that patients with high anxiety may pay more attention to the instruments, which could increase their overall fixation time [7,8].

Eye Tracking in Ergonomics

Eye-tracking technology can also be used to improve ergonomics in dentistry. Ergonomics refers to the design of the workspace and equipment to enable safe and efficient work. Eye-tracking technology can be used to evaluate the ergonomic design of dental workstations and equipment such as dental chairs, dental loupes, and lighting systems. A study by Barabanti and his colleagues found that using eye-tracking technology identified areas of improvement in the ergonomic design of dental workstations [9]. Another study identified applications of eye tracking in dentistry, such as the assessment of ergonomic positions, evaluation of treatment outcomes, and patient communication [10].

Eye tracking in dental education

Multiple studies have investigated the use of eye-tracking technology in dental education, showing promising results in enhancing the learning experience for dental students [10]. The use of eye-tracking technology in dentistry has the potential to become a valuable teaching tool in dental education. Dental students can learn about patient behavior, treatment planning, and communication skills by analyzing visual fixation patterns. Moreover, the eye-tracking device helped massively speed up the practitioner feedback process. Leading to significant improvement and efficiency of the students performing the procedure [11].

Dental Students’ Clinical Skills

One potential application of eye-tracking technology in dentistry is in the training of dental students and novice clinicians. Recent studies have examined the use of eye-tracking technology in dental education, particularly in teaching students clinical procedures. Schittek and colleagues found that the use of eye-tracking technology significantly improved dental students' visual attention during periodontal probing and displayed that this technology has helped students better understand key concepts and techniques involved in dental procedures [12]. Similarly, a review article in 2023 highlighted the potential of eye-tracking technology in dental education, particularly in improving students' clinical skills and reducing errors [13].

Studies have shown that eye-tracking technology can be an effective teaching tool, specifically in dental radiology. Bhadila et al. found that students who received training using eye-tracking technology showed significant improvement in their ability to detect dental caries on radiographs [14]. Another study found that using gaze behavior analysis and feedback during radiology training improved accuracy and efficiency in interpreting radiographs [15].

Communication and Feedback

Eye-tracking technology has also been used to assess the effectiveness of communication between dentists and patients and evaluate treatment satisfaction. Other studies suggest that eye-tracking technology could be a powerful tool for improving dental education by providing efficient and precise feedback on the spot. The technology can be used to identify areas where the student's focus is lacking or where attention is not appropriately devoted during a procedure. Practitioners can then use this information to provide targeted feedback and improve the student's skill level [16,17].

These studies suggest that eye-tracking technology can be a valuable addition to dental education, helping students learn more effectively and efficiently. By tracking students’ gaze and providing instant feedback, this technology has the potential to revolutionize the way dental procedures are taught.

Eye tracking in pediatric dentistry

Over the past decade, eye-tracking technology has been increasingly utilized in various fields to enhance understanding of human behavior and perception. Eye tracking is an emerging tool in pediatric dentistry that can provide valuable insights into children's attention and response during dental procedures.

This technology works by using specialized cameras and software to track the movement and focus of an individual's gaze. When applied in pediatric dentistry, it can show how children look at and respond to different stimuli, such as dental instruments, oral hygiene instructions, or visuals on a screen.

Eye-tracking technology has shown promise in pediatric dentistry as a method of objectively measuring anxiety and attention during dental procedures. With this technology, a small camera tracks the eye movement and records the patient’s gaze patterns. This information can then be analyzed to determine where the patient is looking and for how long, which can provide insight into their level of anxiety or distraction during the dental procedure [18].

One study using eye-tracking technology in pediatric dentistry found that anxious children spent less time looking at the dental equipment and more time gazing at distractions within the room, such as posters on the wall [19]. Another study found that anxious children had a longer fixation duration on dental equipment than non-anxious children [20]. This type of objective data can be helpful in customizing behavioral interventions for patients with dental anxiety.

Another study used eye-tracking technology to investigate the effect of music on the attention and behavior of pediatric patients during dental procedures. The study found that children who listened to music during the procedure had significantly fewer fixations on the dental instruments and more fixations on the ceiling compared to those who did not listen to music. This indicates that music may help distract children from the procedure and make them less focused on the instruments [21].

Additionally, eye-tracking technology may also aid in assessing pain and discomfort during dental procedures. A study examining the use of eye-tracking technology in assessing pain during dental injections found that children who experienced higher levels of pain had a longer fixation duration on the needle [22]. This information can be used to tailor pain management techniques for these patients.

Overall, eye-tracking technology shows potential as a valuable tool in pediatric dentistry for objectively measuring anxiety, attention, and pain during dental procedures. However, further research is needed to validate its usefulness in clinical settings.

One significant advantage of eye-tracking technology is its ability to identify visual preferences and patterns in children. For example, studies have shown that children look more at colorful and animated images while ignoring static or plain images [23]. By identifying these specific preferences, dentists can design educational materials, graphics, or animations that are appealing and engaging to children, thus improving their cooperation and understanding during dental visits.

Moreover, using eye-tracking technology to monitor where the child looks and for how long, dentists can identify moments of high stress or discomfort and adjust their approach accordingly. By adopting a child-centered approach, dentists can effectively manage the child's anxieties, reduce their fear of dental procedures, and improve their overall experience.

Another advantage of eye-tracking technology in pediatric dentistry is its ability to provide objective and quantitative data. Dentists can gain insights into children's cognitive processes and decision-making during dental procedures by collecting and analyzing eye-tracking data. This information can help dentists design interventions tailored to the child's individual needs and preferences, leading to better treatment outcomes and increased patient satisfaction.

In conclusion, eye-tracking technology has excellent potential as a non-invasive and user-friendly tool for improving pediatric dentistry practices. By utilizing eye-tracking data, dentists can gain a deeper understanding of the children's behavioral and visual preferences, evaluate their attention and anxiety levels, and design interventions tailored to their needs. This technology can lead to a more positive dental experience for children and help dental professionals achieve better treatment outcomes.

Eye-tracking technology limitations

Eye-tracking technology has potential benefits for various applications. However, it is important to consider certain limitations related to biometrics. Variations in individual eye characteristics, such as shape and size, as well as the presence of eyeglasses or contact lenses, can affect the accuracy of eye-tracking measurements. Eye pathologies like nystagmus or strabismus can present challenges due to inconsistent eye movements. Environmental factors, including lighting conditions and glare, can also impact the quality of eye-tracking data. Furthermore, participant cooperation and fatigue play a role in obtaining reliable results. Calibration variability and the limited field of view of eye-tracking systems further contribute to potential inaccuracies [2-4].

When utilizing eye-tracking technology in dentistry, It is essential to acknowledge these limitations and work towards improving the technology to mitigate these constraints. A comprehensive review conducted by Shafi et al. identified the need for more consistent standards in study designs when using eye-tracking technology. The study stated various factors such as the types of stimuli used, regions of interest, and recording equipment which can impact the results and limit the comparability between studies [24].

Another limitation of eye-tracking research is the small sample sizes typically used in studies, leading to limited generalizability of the results. Additionally, some studies may be limited by using artificial stimuli or non-dental tasks, which may not perfectly replicate the conditions of an actual dental appointment. Despite its potential to provide valuable insights into patient behavior and attention patterns during dental procedures, its adoption has been slow due to the cost associated with the technology and the need for specialized equipment, software, and professional training [24].

In the future, eye-tracking data can be combined with artificial intelligence (AI) and machine learning algorithms to develop predictive models for diagnosing dental conditions, assessing treatment outcomes, and optimizing treatment plans. This could lead to more accurate and efficient dental care delivery.

## Conclusions

In conclusion, it is indicated there is a need for further research into the clinical implications of eye tracking in dentistry, including how the results can be translated into actionable recommendations for dental practitioners. Despite these limitations, however, eye-tracking technology holds promise for enhancing patient care and clinical outcomes in dentistry. Continued research and development in this field have the potential to improve our future understanding of patient’s perceptions and the quality of dental care provided. Therefore, eye-tracking technology represents an exciting opportunity to advance dental education, research, and clinical practice.
